# Metabolomics for Evaluating Flavor-Associated Metabolites in Plant-Based Products

**DOI:** 10.3390/metabo10050197

**Published:** 2020-05-15

**Authors:** Shruti Pavagadhi, Sanjay Swarup

**Affiliations:** 1 Department of Biological Sciences, National University of Singapore, Singapore 117558, Singapore; pavagadhis@nus.edu.sg; 2 Singapore Centre for Environmental Life Sciences Engineering, National University of Singapore, Singapore 117456, Singapore; 3 NUS Environmental Research Institute, National University of Singapore, Singapore 117411, Singapore

**Keywords:** plant-based diets, plant-based products, metabolomics, sensory attributes, flavor

## Abstract

Plant-based diets (PBDs) are associated with environmental benefits, human health promotion and animal welfare. There is a worldwide shift towards PBDs, evident from the increased global demand for fresh plant-based products (PBPs). Such shifts in dietary preferences accompanied by evolving food palates, create opportunities to leverage technological advancements and strict quality controls in developing PBPs that can drive consumer acceptance. Flavor, color and texture are important sensory attributes of a food product and, have the largest influence on consumer appeal and acceptance. Among these, flavor is considered the most dominating quality attribute that significantly affects overall eating experience. Current state-of-art technologies rely on physicochemical estimations and sensory-based tests to assess flavor-related attributes in fresh PBPs. However, these methodologies often do not provide any indication about the metabolic features associated with unique flavor profiles and, consequently, can be used in a limited way to define the quality attributes of PBPs. To this end, a systematic understanding of metabolites that contribute to the flavor profiles of PBPs is warranted to complement the existing methodologies. This review will discuss the use of metabolomics for evaluating flavor-associated metabolites in fresh PBPs at post-harvest stage, alongside its applications for quality assessment and grading. We will summarize the current research in this area, discuss technical challenges and considerations pertaining to sampling and analytical techniques, as well as s provide future perspectives and directions for government organizations, industries and other stakeholders associated with the quality assessment of fresh PBPs.

## 1. Introduction

### 1.1. Global Food Palates: Shifts Towards Sustainable Future Food

Increasing urbanization, rising per capita incomes and affordability are shaping the way our food is produced and consumed globally. The associated changes in lifestyle are influencing the composition of food baskets, food consumption patterns and behaviors [1,2,3,4]. With the advent of digitalization and increased access to information, consumers are becoming more cognizant about food and its sources [5]. There is increasing focus on well-being and shifts in consumer preferences toward foods that are grown sustainably. Consequently, plant-based diets (PBDs) are gaining popularity owing to their numerous environmental and human health benefits [6].

### 1.2. Plant-Based Diets: What Do We Know?

Diet refers to a lifestyle adopted by an individual, and largely relates to an eating plan and regimen for habitual nourishment. With PBD, an individual relies on plant-based products (PBPs) for his/her daily nutritional needs. Typical PBDs maximize the consumption of nutrient-rich plant foods while minimizing processed foods, oils, and animal foods (including dairy products and eggs) [7]. It is pertinent to note that at present, there are varying opinions in the scientific community about idealistic PBDs. However, there is a general cognizance that PBDs are associated with a multitude of human and environmental health benefits. Some epidemiological and interventional human studies have suggested that PBDs exert beneficial health effects against obesity-related metabolic dysfunction, type 2 diabetes mellitus and chronic low-grade inflammation [8,9,10]. Furthermore, the production of PBDs tend to be less resource-intensive and more environmentally friendly for various reasons, including lowered levels of greenhouse gas emissions (GHGEs), in comparison to raising animals for human consumption [11].

As most PBDs rely heavily on plant-based products (PBPs), there will be an increased global demand for PBPs to meet the changing consumer preferences. For the purpose of this review, the scope will be limited to fresh PBPs at the post-harvest stage, where the produce makes its first entry for quality assessments.

### 1.3. PBPs: Nutritional and Sensory Properties

PBPs comprise of vegetables, fruits, lentils, grains, legumes, nuts and seeds. They offer a myriad of nutritional and functional benefits for human health promotion. Apart from macronutrients and micronutrients, many of these PBPs provide a range of bioactive compounds to combat inflammation, strengthen antioxidant defenses, and general immune system [12,13,14].

A considerable fraction of bioactive compounds/metabolites in PBPs, such as pigments, phytochemicals and other secondary metabolites, contribute to the sensory properties of fresh PBPs. Flavor, color and texture together contribute to the overall eating experience associated with PBPs, and are often a deterministic factor in influencing consumer acceptance. Among these three sensory properties, flavor often has the highest influence on consumer acceptance and behavior. Apart from being a critical quality attribute, flavor also provides valuable information about the nutritional quality of the food [15]. While consumers generally recognize flavor as the most dominant quality attribute for certain PBPs such as fruits and vegetables, it is the interaction of flavor and texture that has a significant effect on consumer acceptance of PBPs [16]. However, for the purpose of this review, we will focus on flavor-related attributes of fresh PBPs. Flavor is perceived primarily by the sense of taste and olfaction (aromatics/aroma) [17]. Aroma and taste receptors, located in the nose and mouth, respectively, are responsible for distinct flavor recognition. It is generally accepted that olfactory stimuli (aroma metabolites) contribute significantly to the flavor experience of most food products. The unique taste sensations and aroma associated with PBPs come from a complex mixture of compounds that belong to different chemical classes. They originate from the primary and secondary metabolism in PBPs and are generally bioactive, with aroma metabolites being volatile in nature while, the taste metabolites often being non-volatile. Both the volatile and non-volatile bioactive fraction in PBPs, such as phenols, flavonoids, isoflavones, terpenes, and glucosinolates, contribute to bitter, acidic, or astringent flavor profiles [18,19]. The presence of these bioactive compounds is an intrinsic property of PBPs, and their synthesis is often influenced by multiple genetic and environmental factors [20,21,22]. Considering the diverse nature of these bioactive compounds and their contribution to the flavor of fresh PBPs, an inclusive approach for their quality assessment at the post-harvest stage is valuable for entire supply chain management. The significance of including a detailed characterization of bioactive compounds for quality assessment has received considerable attention for certain processed food products [23,24,25]. However, quality assessment for fresh PBPs at the post-harvest stage mainly relies on conventional techniques, as discussed in the next section.

## 2. Quality Assessment of PBPs

### 2.1. Post Harvest Handling: Current State-of-Art Technologies for Flavor Related Attributes

At present, the post-harvest quality assessment of fresh PBPs is effectively regulated for attributes related to food safety/human health risk (heavy metals, chemical contamination, microbiological), but loosely regulated for attributes associated with consumer acceptance and eating experience. These regulations are imposed both at international and national levels, as well as within the individual supply chains [26]. Current quality assessment parameters do not effectively inform on the kind of metabolites or chemical compounds that are responsible for the unique flavor profiles of fresh PBPs. However, this could be particularly important for formulating new products in this domain, keeping in mind the changing consumption trends and evolving flavor preferences.

For any PBP, the relative importance of a quality attribute depends on the commodity and its end-use [27]. In general, the post-harvest handling steps for PBPs include identification of the key quality attributes from food safety/human health-related risks (minimum statutory requirements), followed by establishing quality control/quality assurance (QA/QC) procedures to (i) maintain acceptable quality level for the consumer; and (ii) ensure that minimum quality standards are met.

The quality assessment of fresh PBPs routinely involves sensory and instrumental methods. In general, sensory methods are used for developing new products and determining product standards, while instrumental methods fare better in assessing the quality of the fresh PBPs on a routine basis [28]. Sensory evaluation is usually performed by a trained sensory panel, and it has two components: the analytical component, which is used to detect differences in products, and affective measurements, which determine preference. Instrumental measurements, on the other hand, focus on the chemical and physical characteristics of PBPs, and encompass a wide range of techniques to determine flavor attributes. For example, a hydrometer that can detect total soluble solids is often used to determine sugar levels while, pH meter is used to measure the level of sourness in food products [28].

### 2.2. Gaps in Current Technologies and Need for Complementary Approaches

Instrumental techniques aimed at evaluating the physical and chemical characteristics of PBPs are advantageous as they: (i) provide high accuracy and great precision; (ii) are often more sensitive to small differences between samples, which assist in determining quality trends; and (iii) they are high-throughput and are often available in semi-automated and automated formats [29]. However, the physicochemical characteristics of PBPs have little relevance to consumer acceptability and thus, the results can be used in a limited way to define the quality attributes of PBPs [30]. For this purpose, sensory evaluation is often recommended to accurately assess the quality attributes of fresh PBPs. Sensory evaluation also has certain disadvantages as it requires a trained sensory panel and it is often time consuming, laborious and challenging.

To complement and extend the repertoire of the existing methodologies, detailed and quantitative analyses to measure flavor-associated metabolites are warranted. Integrating such technologies in current quality assessment of fresh PBPs will (i) ensure product uniformity; (ii) strengthen consumer acceptability for PBDs and PBPs in general; (iii) complement current assessment platforms for quality and food safety of fresh PBPs; and (iv) aid in determining maturity and degree of ripening of PBPs at the post-harvest stage.

## 3. Metabolite Fingerprinting for Quality Assessment of PBPs

### 3.1. Metabolomics in Agri-Food Sector: Current Practices

Metabolomics allows for studying multiple small molecules or metabolites in a cell, tissue or organism. It is defined as the comprehensive characterization of small molecules present in a biological sample [31,32,33]. Metabolomics routinely utilizes sophisticated and high-throughput analytical platforms such as gas chromatography and liquid chromatography–mass spectrometry (GC–MS and LC–MS) and nuclear magnetic resonance (NMR) spectroscopy [34]. With the advent of chemometrics and advanced analytical platforms, metabolomics has greatly facilitated our understanding of the global metabolome and pathway networks [35]. Metabolomics approaches involve untargeted or targeted analyses, and the selection of the approach is largely dependent on the experimental question and expected outcomes [36]. Untargeted analyses utilize an unbiased profiling or metabolic fingerprinting approach focused on uncovering the global metabolome to evaluate diverse chemical classes of metabolites associated with different pathways. On the other hand, targeted analyses rely on a priori knowledge of the class of metabolites or pathways that are of interest [37]. However, the combination of these analyses is often required to obtain complete information of interest.

Over the past few decades, metabolomics has been extensively applied to various fields of science owing to new developments in analytical instrumentation and data-analytics platforms [38,39,40,41]. Although still in their infancy, metabolomics-based approaches have gained significant interest in the agri-food sector for a diversity of applications including food processing, quality control, plant breeding for improved crop varieties and product development [42,43]. However, at present, metabolomics-based approaches have not been adopted by the regulatory agencies for food quality assessment, although in some cases they have been found to be efficient, with clear benefits over conventional methods. For instance, metabolomics-based approaches have proved valuable to the food industry for the aroma analysis of fresh and processed PBPs [44,45,46,47]. It is pertinent to note that most of the current research in food metabolomics is focused on evaluating various quality attributes of processed/semi-processed food products. Efforts in the area of fresh produce are mostly restricted to economically important PBPs or PBPs grown for specific end-use [45].

### 3.2. Metabolomics for Evaluating Flavor Associated Metabolites in Fresh PBPs

Within the agri-food sector, several diverse areas utilize metabolomics approaches for a variety of applications, as discussed in the previous Section 3.1. One such application involves evaluating the flavor-associated metabolites in fresh PBPs, which are determined by their biochemical composition. As stated in earlier Section 3.1, flavor has the largest influence on consumer behavior and consumption pattern [15], and consequently, most of the research efforts in this domain are catered towards determining the flavor-related metabolites in PBPs. Perception of flavor involves both volatile aroma metabolites as well as non-volatile taste metabolites which belong to different classes.

#### 3.2.1. Aroma Associated Metabolites

In fresh PBPs, a diverse set of volatile chemical compounds contribute to their natural aroma, increasing the complexity of these aroma-associated metabolites. This complexity is further compounded as the volatile compounds interact with each other to create a unique aroma profile for PBPs, which is not merely a sum of the volatile compounds present in them. To date, more than 7000 volatile compounds have been identified in foods, however, a relatively small number (300–400), in specific abundance and ratio, determine the characteristic aroma of the product [48,49].

There are several known classes of volatile aroma metabolites that contribute to the unique flavor of fresh PBPs, such as esters, alcohols, aldehydes, ketones, lactones, terpenoids and apocarotenoids. However, derivatives of amino acids, lipids, phenolic acids and sesquiterpenes are known to be the most important aroma-associated metabolites in PBPs [50]. In certain PBPs, especially fruits and vegetables, sulphurous compounds and derivatives also contribute to their distinct aroma profiles [50]. Volatile aroma metabolites associated with fresh PBPs are generally derived from phytonutrients belonging to fatty acids, amino acids, carotenoids and terpenoid classes [15,51] through a limited number of major biochemical pathways [52]. These pathways are mainly involved in the synthesis of the backbone, while the diversity of these volatiles is achieved via additional chain modification steps and further transformations. Fatty-acid derived volatiles such as alcohols, esters, ketones, acids and lactones form important character-impact aroma compounds that are responsible for flavors of fresh fruits mainly synthesized through *α*-oxidation, *β*-oxidation and the lipoxygenase pathway [53].

Similarly, amino acid-derived volatile compounds are produced either through amino-acid precursors (direct) or through acyl-coAs (indirect) and they mainly belong to alcohols, esters, and vegetables. Amino acid-derived volatiles represent dominant classes in PBPs, specifically fruits, vegetables, and grains [15,19,54,55]. For instance, the amino acid proline is the nitrogen precursor for 2-acetyl-1-pyrroline, a volatile compound that is associated with the aroma of certain rice varieties. Similarly, methionine and tryptophan are involved in side-chain modifications of sulphur containing glucosinolates, which result in volatile degradation products, namely isothiocyanates, that contribute to the characteristic aroma associated with *Brassica* genus [56]. Terpenoids make up the largest class of plant secondary metabolites, many of them being volatile in nature, that contribute to the aroma of fresh PBPs. Hemiterpenes (C5), monoterpenes (C10), sesquiterpenes (C15), homoterpenes (C11 and C16), and some diterpenes (C20) have higher vapor pressure, allowing their release into the surrounding atmosphere and volatilize. All the terpenoids are derived from the universal C5 precursor isopentenyl diphosphate (IPP) and its allylic isomer dimethylallyl diphosphate (DMAPP) [57]. Many terpene volatiles are direct products of terpene biosynthesis enzymes, while some are derived through modifications and additional transformations of primary terpene skeletons, mainly via hydroxylation, dehydrogenation, acylation. For instance, hydroxylation of limonene results in the formation of *trans*-isopiperitenol and *trans*-carveol through different catalyzing enzymes and these hydroxylated terpenes are associated with characteristic flavor of certain PBPs [58,59]. Similarly, acetylation of certain terpenes like geraniol results in the formation of geranyl acetate, which has a pleasant fruity aroma and is found in many PBPs. Apart from fatty acid, amino acid and terpenoid pathways, carotenoid pathways represent another major class of volatiles in PBPs. Carotenoid derivatives mainly derived via the oxidation cleavage of carotenoids result in the formation of volatile apocarotenoid derivatives [60]. These volatiles contribute to the aroma of several vegetables and fruits [61].

In the past few years, several research studies have exploited metabolomics approaches to evaluate these diverse classes of aroma metabolites in a variety of fresh PBPs. We provide a representative summary for some of these aroma metabolites in selected fresh PBPs (Table 1). Studies have utilized different kinds of analytical platforms and extraction approaches to analyze chemical classes that contribute to the unique aroma and flavor of fresh PBPs, as summarized in Table 1.

#### 3.2.2. Taste Associated Metabolites

Taste metabolites are quite closely linked with aroma metabolites. These metabolites are generally non-volatile in nature, and they contribute to the flavor profiles by enhancing the gustatory experience via accentuation of the volatile aroma metabolites. There are five kinds of taste perceptions, namely, sweet, salty, bitter, sour and umami. Different chemical classes of metabolites contribute to the taste sensation in PBPs. Sweetness generally comes from sugars, including sucrose, glucose, and fructose. The levels of these sugars are often influenced by genetic and environmental factors and are highly associated with the degree of ripening. A wide variety of PBPs, including fruits and vegetables, have varying levels of these sugars and their biosynthesis is genetically controlled and regulated. Sourness is derived from acids such as malic, citric, and oxalic acid. Bitterness is often associated with presence of polyphenols, alkaloids, tannins, certain glycosides, or peptides. For example, tannins provide the bitter notes and complements the flavor of several PBPs including tea and immature berries [77,78]. Among polyphenols, the taste of bitterness and tactile sensation are often associated with flavonoid phenols, including flavanols and flavonols. Some of the metabolites from these families such as proanthocyanidins or condensed tannins are abundant in wine and tea [79]. Salty and umami tastes are not common in PBPs. Among the taste sensations in PBPs, bitterness is the most complex, as structurally diverse chemical compounds/metabolites can elicit a single bitter taste, which suggests that multiple mechanisms are responsible for the perception and transduction of bitterness. It is also pertinent to note that small changes in chemical structure can transform bitter compounds to sweet or vice versa. Scientific evidence also suggests that bitter and sweet tastes, when present together, can enhance, or suppress each other [80]. In recent years, research initiatives have been directed towards evaluating metabolites that contribute to different taste sensations in a variety of fresh PBPs including fruits and vegetables. Among the taste-associated metabolites, polyphenols are studied extensively among a wide range of PBPs. Polyphenols are a ubiquitous class of non-volatile plant secondary metabolites and apart their sensory attributes, they are also known for their anti-inflammatory and other metabolic effects [81,82,83,84]. Polyphenols are biosynthesized by plants for chemical defense against predators and among them, class of flavonoids are associated with taste sensations in PBPs. Most of them contribute to a bitter taste in PBPs [77,78], but owing to their health benefits, several efforts are directed towards debittering the food products to increase its consumer acceptance [81]. This interest could also be partly responsible in the research impetus on understanding composition of polyphenols and their sensory attributes in PBPs. Representative research studies reporting diverse classes of polyphenols in various PBPs using various analytical platforms are summarized in Table 2.

Currently, for flavor-associated metabolite profiling, several extraction techniques and analytical platforms are employed to capture the analytes of interest, which are discussed in the next section. The number of studies reported in this area are progressively increasing with the advent of rapidly evolving analytical platforms, curated databases, automated sample, and liquid handling systems. There is a scientific cognizance about the potential of metabolomics for this growing field, although it has not been widely adopted for routine quality assessment due to a variety of factors including sampling considerations and technical challenges.

### 3.3. Sampling and Other Considerations for Metabolomics

As detailed in the earlier Section 3.2, the use of metabolomics in evaluating flavor attributes of fresh PBPs is gaining considerable interest in the scientific community. However, its widespread adoption by agri-food related sectors and regulatory agencies would require streamlining (i) sampling protocols; (ii) pre-concentration and extraction procedures; and (iii) analytical platforms and approaches. We describe below these three important points for consideration in order to successfully employ metabolomics for evaluating flavor-associated metabolites in fresh PBPs.

(i) Sampling protocols: As the biosynthesis of flavor-associated metabolites in fresh PBPs is often influenced by several genetic and environmental factors [87], sampling protocols are a critical step in determining true readouts. Environmental factors including farm/management practices, degree of maturity and post-harvest handling will affect the abundance of these bioactive metabolites in the fresh PBPs [88]. Apart from the environmental factors, the nature of these metabolites and their chemistries will also influence the sampling protocols and operational procedures as some metabolites are found in bound form, while others are released only upon tissue disruption. For instance, certain aroma metabolites are only released upon cell disruption when enzymes and their corresponding substrates interact [89]. However, some aroma compounds are bound to sugars as glycosides or glucosinolates [90] and odorous aglycones could be released from the sugar moiety during post-harvest stages. Hence, it is pertinent to adopt sampling protocols that can capture the metabolites of interest in a PBP. To simplify, protocols can be standardized for certain families of PBPs, which are known to have similar metabolite classes. For instance, members of *Brassica* genus (such as broccoli, cabbage, kale) are known to contain glucosinolates (GSLs, sulphur rich secondary metabolites) contributing to their bitter taste and unique aroma [91], and sampling protocols can be standardized across members of this genus for efficient capture of GSLs. Alternatively, protocols can be standardized across different PBPs for the same families of metabolites, such as benzenoids, alcohols and esters. Sampling time-points are equally important, as it is known that PBPs have varying levels and kinds of metabolites at different growth and maturity stages. For instance, it is known that the growth stage has an influence on specific GSLs composition and content among members from *Brassica* genus [92]. Similarly, anthocyanins are also regulated differently at different developmental and ripening stages [93].
(ii) Pre-processing and extraction procedures: Apart from sampling protocols, the choice and selection of pre-processing and extraction procedures are equally important due to the thermolabile nature and trace concentrations of these metabolites in fresh PBPs. Extraction procedures largely depend on (i) the nature and chemistry of metabolites (polar/non-polar; volatile/non-volatile); (ii) the thermal stability and sensitivity; and (iii) their occurrence and subsequent release. A variety of methods are prescribed for the extraction and characterization of metabolites linked to the flavor properties of fresh PBPs. Due to the volatile nature of a variety of aroma-metabolites, headspace analyses involving the gas phase in equilibrium with PBPs are commonly utilized for flavor analyses. The headspace-solid phase microextraction (HS-SPME) is notable for being sensitive, solvent-free and has been successfully employed for flavor extraction of fresh PBPs [94,95]. SPME fiber coatings with different polarities are often required for effective capture of aroma-metabolites with varying chemistries and affinities [96]. However, the limitations of SPME have been pointed out for the quantitation of certain volatile classes of aroma-metabolites [97]. Other techniques used for capturing volatile and semi-volatile metabolites from PBPs are solvent-less enrichment techniques, such as stir bar sorptive extraction (SBSE) [98] and headspace sorptive extraction, (HSSE) wherein stir bar (covered in polysiloxane) is exposed to the sample (either in gaseous or liquid sample media). After extraction, compounds are thermally desorbed before analyses. Extraction techniques assisted by solvents and thermal distillation have been utilized for certain classes of organosulphur metabolites. Steam distillation (SD), simultaneous distillation and solvent extraction (SDE), and solid-phase trapping solvent extraction (SPTE) are used to characterize sulphur-rich aroma-metabolites in certain fresh PBPs such as garlic and onion [98]. Similarly, liquid–liquid extraction (LLE) and solvent-assisted flavor evaporation (SAFE) are used as preferred extraction techniques for furan derivatives that contribute to flavor profiles of certain PBPs [99]. It is pertinent to note here that several extraction techniques have been evaluated based on trapping, capture and dissolution of metabolites to enhance metabolite coverage from plant matrices.
(iii) Analytical platforms and approaches: As seen in the previous section, analytical approaches and platforms are also dependent on the metabolites of interest. GC-O or GC-MS (gas-chromatography-olfactory/gas-chromatography mass-spectrometry) are routinely employed for the detection of aroma- and odor-producing metabolites [63,65,69]. In olfactometric techniques, the nose is used as a GC detector. The GC system can be set up with the column split, and a portion of the effluent goes to the sniffing port and the remainder is fed to the GC detector (FID or an MS detector). GC-O produces an aromagram, which lists the odor character of each peak in a GC run. This method is dependent on the analyst and his sensory perception and, hence, this is a powerful technique which can bridge the conventional sensory evaluation and panel tests with more quantitative information. GC-O can be employed to distinguish between characteristic and off-odors in fresh PBPs, which will assist in quality assessment in terms of food safety and consumer acceptability. While GC-O is more to detect odor and aroma-metabolites, when it is paired with MS detector, it can be used as an identification tool to characterize and quantitate certain metabolites of interest [100]. Other instrumental methods used include NMR and LC-MS. LC-MS platforms are mainly restricted for non-volatile classes of metabolites [82,83,84] such as organic acids, sugars and certain polyphenols which contribute to characteristic taste notes in fresh PBPs.

Lately, biosensors (such as electronic noses and electronic tongues) based on pattern recognition of flavor and aroma metabolites have been developed that can crudely mimic the human taste and olfactory receptors and their communication with the human brain [101,102]. These electronic noses (e-noses) and electronic tongues (e-tongues) do not generate information on sample composition but provide a digital fingerprint through pattern recognition. These devices are capable of mimicking human smell and taste sensors based on previous exposure leading to pattern recognition through neural networks. This is useful for routine post-harvest quality assessment of fresh PBPs to evaluate produce for optimum flavor attributes. For instance, it can be used to evaluate effects on storage conditions on quality of fresh PBPs [103]. Recently, e-noses have been utilized for diverse PBPs (especially fruits and vegetables) to evaluate volatile metabolites that are associated with flavor and/or post-harvest quality of PBPs [104,105,106]. Most often, these sensors have been used in combination with GC-O/ GC-MS techniques with or without sensory analyses, as summarized in Table 3.

To summarize, reliable and credible estimations of metabolites corresponding to flavor-related sensory attributes in PBPs require careful sampling strategies, thorough pre-processing and extraction procedures followed by robust analytical platforms.

### 3.4. Metabolomics and Quality Assessment of PBPs

The quality of the fresh PBPs in terms of their nutritive value and flavor profiles is essentially driven by their biochemical composition. Biochemical composition is also a key factor in determining other important properties of fresh PBPs such as shelf life, nutritional stability, and economic value. New tools are required to define “quality” to include more quantitative information about the biochemical composition of food, as consumers’ expectations continue to grow with respect to food quality and safety [114]. Meanwhile, current quality assessment relies heavily on classical methodologies which can largely inform general consumer acceptability, but they lack the ability to provide detailed information on biochemical composition or metabolites that correspond to unique flavors of fresh PBPs. To this end, metabolomics can pave the way for decoding the composition and nature of flavor-associated metabolites in fresh PBPs, which can open avenues for further improvements of PBPs [115,116]. As such, the scope of metabolomics in this domain extends beyond just quality assessment for flavor-associated metabolites; it can be further utilized for (i) biomarker-detection related to food safety; (ii) development of new crops with better genetic traits; (iii) determination of food contaminants/adulterants; and (iv) new investigations on food bioactivities [117,118,119].

Although a distinct research area on food metabolomics has been established in the scientific community in relation to the application of metabolomics in food system processes [119,120] from farm to consumers, its widespread adoption comes with certain unparalleled challenges (as discussed in Section 3.3). These challenges are often compounded by the nature of the food metabolome, which is complex and variable in nature as thousands of metabolites are present in fresh PBPs with varying polarities and chemistries [121]. Measuring and quantifying the metabolome that best represents the flavor profiles of fresh PBPs can pose analytical challenges as it may not be possible to detect all of them in a single analysis. To this end, utilizing multiple analytical techniques and approaches is often recommended in food metabolomics which can complement each other and provide a wider coverage [122,123]. In addition to this, the food matrix of PBPs will also affect the detection and quantification of compounds that are present at very low concentrations in fresh PBPs or are present in bound forms and unstable forms (as discussed in Section 3.3). Apart from these analytical and sampling-related hurdles, there are certain challenges at downstream data processing and integration with current quality assessment methodologies, and these will be discussed in the next section.

## 4. Flavor Evaluation of Fresh PBPs: Way Forward

As discussed in Section 3.3 and Section 3.4, there is an immediate need to extend and complement the current repertoire of sensory-based and coarse instrumental estimations to evaluate the flavor- associated metabolites in fresh PBPs. This need is fueled by several socio-economic and psychological factors that have been discussed in the earlier section (Section 1.1 and Section 1.2). Against this background, the current quality assessment methodologies for fresh PBPs will need to be more inclusive of systematic metabolic estimations for flavor attributes in fresh PBPs. Metabolomics can prove to be a valuable tool in this regard, however, utilizing this technique with other routine quality assessment methodologies will require careful considerations at multiple levels. Additionally, it is pertinent to note here that although metabolomics can provide useful biochemical insights about flavor-associated metabolites in PBPs, it cannot provide any information on the human perception of food flavors, which is often influenced by physiological, psychological, genetics and other associated socio-cultural factors [124,125,126]. These factors contribute to the inter-individual variation and cause stark differences in perception of these metabolites by various population groups. To account for these differences, sensory-based tests will remain critical to obtain holistic understanding on consumer acceptance and behavior.

**Figure 1:**
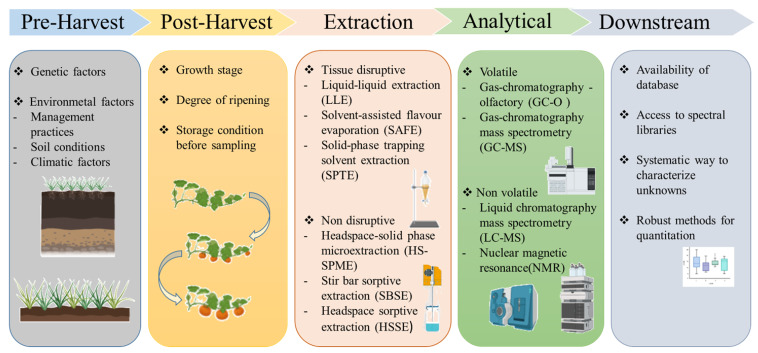
Considerations for utilizing metabolomics for evaluating flavor-associated metabolites in fresh PBPs. Here, we describe the various factors that will have an effect on metabolite estimations in fresh PBPs.

**Figure 2:**
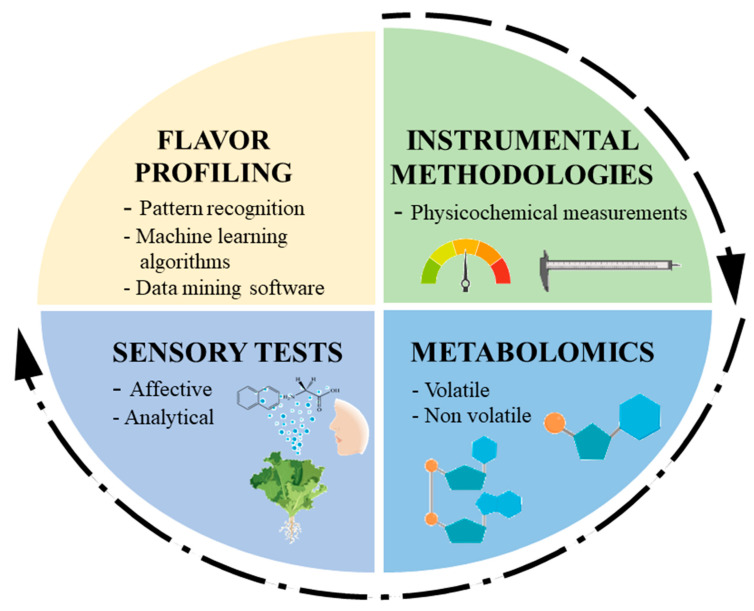
Framework for integrating metabolomics with current state-of-the-art technologies for the organoleptic evaluation of fresh PBPs. While physicochemical measurements are coarse-scale estimations, metabolomics and sensory-based tests serve as fine-scale estimations to achieve a holistic flavor profiling of fresh PBPs. Data integration platforms would play a crucial role to achieve seamless data stitching for meaningful insights.

To harness the potential of metabolomics for evaluating flavor-associated metabolites in PBPs, it is important to keep in mind that both pre and post-harvest procedures including the extraction and analysis of metabolites will have great bearing on the observed results (Figure 1). In order to complement the existing sensory-based and instrumental measurements, a clear interface and seamless integration has to be established between the pre and post-harvest procedure in order to s to ensure aa smooth workflow for rapid quality assessment of fresh PBPs (Figure 2).

Integrating metabolomics with the current state-of-the-art technologies for quality assessment will require synchronized efforts at several points—all the way from data collection to data analyses. To maximize the potential of metabolomics approaches, data collation from various platforms along with careful data interpretation will undoubtedly play a key role. This can lead to several other technical challenges, depending upon the category of PBPs in question, and the nature of information required. Some of the technical challenges could be related to the availability of (i) the right instrumental platform or extraction protocols for metabolites of interest; (ii) reference databases and spectral libraries for matching interesting metabolic features in PBPs; and (iii) the complete metabolome or databank for the plant source in question.

Owing to the rapid developments in extraction and analytical methodologies, several options are available to analyze metabolites with varying chemistries, thereby increasing the global metabolite coverage. With these developments, the first technical hurdle can be conquered with few rounds of trials and optimization. However, the second and third technical challenges pose the greatest difficulty, not just for the agri-food domain, but also for other scientific domains, as a lack of reference databases and metabolome information makes it difficult to interpret the data and obtain meaningful insights. Lately, several curated databases have been made available in the plant domain specifically for diverse phytochemicals and bioactive metabolites to help the research community [127,128]. As the plant metabolome is highly diverse, with thousands of metabolites, it presents a laborious and technically challenging task to annotate every single metabolite. To overcome this challenge, efforts can be strategized towards the identification of candidate/marker metabolic members from different classes that can best represent the specific PBPs. This would eliminate the need to identify each metabolite and, at the same time, will serve as reference for rapid screening of PBPs based on presence of certain key metabolite classes. The choice and selection of such metabolite classes would depend upon the type of PBPs and their ultimate end-use. Monitoring glucosinolates (sulphur containing metabolites) in members from *Brassica* genus can be a classical example for this, as these metabolites are (i) unique to *Brassica* family members; (ii) associated with the flavor attributes of these plant types; and (iii) known for their human health benefits. Similarly, eucalyptols (cyclic ether, monoterpenoids) are unique to members of the *Myrtaceae* family and they are known for imparting a mint-like aroma and spicy taste notes. Other examples include PBPs from *Amaryllidaceae* that contain S-alk(en)yl-l-cysteine sulfoxides. Another way to approach this challenge will be to generate reference metabolic fingerprints of PBPs and utilize machine learning-based algorithms for pattern recognitions and high-throughput screening. This approach relies on the premise that if sampling, extraction and analytical conditions are kept the same, metabolic fingerprint from two PBPs samples of same type would be identical or similar to a large extent. However, this approach should be utilized as a fast screen and for more quantitative information, in-depth analyses are recommended. Apart from these technical challenges, a systematic method to integrate and collate the data from various platforms is warranted to maximize the potential of multi-platforms in the sensory evaluation of fresh PBPs.

A significant improvement has been achieved in recent years in data integration and chemometrics pipelines, making it easier to obtain integrated biological outputs from different platforms. In recent years, numerous tools have been developed, written in most used programming languages such as Python, R, and Matlab^®^ to aid in metabolomics data curation and management [127]. Additionally, several platforms are being made available for sharing scripts and workflows through open-access repositories (Github, StackOverflow). Interactive and intuitive data integration workflows are being developed that have adopted artificial intelligence (AI) and machine learning (ML) approaches [129,130]. Data integration platforms that combine e-noses and e-tongues with high resolution MS and analytical instrumentation could be a way to logically bridge current gaps between human-based sensory tests and metabolic estimations. Although these artificial sensory techniques cannot integrate taste and smell as can be done by the human sensory system, they can generate reliably consistent data in a high-throughput format. With the availability of the requisite computational power, it is possible to integrate such modular information from these artificial sensors to obtain meaningful insights [131,132].

This will be particularly resourceful for innovations and new product developments in this domain as we continue to witness intense reformation and diversification of food palates globally. In addition, integrating these platforms with trained artificial intelligence can further uplift them to smart sensing platforms that can, to an extent, also predict emerging food safety threats in terms of adulterants and/or pathogens. To make this a real scenario, coordinated efforts and synchronized response will be required from several stakeholders working in this evolving domain.

## 5. Conclusions

To conclude, utilizing metabolomics for evaluating flavor-associated metabolites in PBPs would likely become a necessity in coming years and it will see multiple applications from product authenticity, quality assessment, new product development and enhanced food safety.

## Figures and Tables

**Table 1:** Aroma-related metabolites determined using different analytical platforms in fresh plant-based products (PBPs). A representative summary of recent research studies in this area.

S.no	Metabolites Classes	PBP Type	Analytical Platform	References
1	Esters, alcohols, aldehydes, ketones, lactones, terpenoids, sulphur compounds	Melons (*Cucumis melo* L.)	GC-MS GC-O	[62]
2	Alcohols, acids, and carbonyl compounds, terpenoids and norisoprenoids, furan, phenols and phenylpropanoids, benzonoids, furans	Kiwifruit (*Actinidia deliciosa*)	GC-O	[52]
3	Monoterpene hydrocarbons and oxides, sesquiterpenes, aldehydes, alcohols, esters	Japanese citrus fruit (*Citrus nagato-yuzukichi Tanaka*)	GC-MS	[63]
4	Esters, alcohol, fatty acid esters, carboxylic acid esters	Pear fruit (*Pyrus communis*)	HRGC-C/P-IRMS	[64]
5	Esters, aldehydes, alcohol, benzenic derivatives, ethers	Ambul Banana (*Musa acuminata, AAB*)	GC-MS	[65]
6	Aldehydes and alcohols	Potato (*Solanum tuberosum*)	GC-FID	[66]
7	Aliphatic acids, aldehydes, alcohols, Oxygenated and nonoxygenated monoterpenes, phenolic derivatives, nor-isoprenes	Tomato (*Solanum lycopersicum*)	GC	[67]
8	C8-C9 unsaturated aldehydes and ketones	Oat (*Avena sativa*)	GC-MS, GC-O	[68]
9	Ketones, alcohols, esters, and heterocycle compounds	Intermediate wheatgrass (*Thinopyrum intermedium*)	GC-MS-O	[69]
10	Unsaturated hydrocarbons, carboxylic acid esters, phenol ethers	Rice (*Oryza sativa*)	GCGC-TOFMS	[70]
11	Alcohols, aldehydes, ketones, nitrogen-compounds, Straight- and branched-chain hydrocarbons	Jasmine brown rice (*Oryza sativa*)	GC-MS	[71]
12	Ketones, aldehydes, pyrazines, alcohols, aromatic hydrocarbons, furans, pyrroles, terpenes, and acids	Turkish Tombul Hazelnut (*Corylus avellana* L.)	GC-MS	[72]
13	Alcohols, aldehydes, esters, benzene derivates, linear hydrocarbons, ketones furans	Dark Black Walnut (*Juglans nigra*)	GCMS	[73]
14	Monoterpenes	Pistachio nuts (*Pistacia vera* L.)	GC-MS	[74]
15	Pyrazines, aldehydes, alcohols, ketones, esters, carbonic acids, furan derivatives, pyrroles, pyridines, pyran derivatives, hydrocarbons, phenols, sulphur compounds, lactones	Wheat flour bread (*Triticum aestivum*)	GC-MS	[75]
16	Aliphatic hydrocarbons, monoterpenes and such	Walnuts (*Juglans regia* L.)	GC–MS	[76]

**Table 2:** Taste-related metabolites determined using different analytical platforms in fresh PBPs. A representative summary of recent research studies in this area.

S.no	Metabolites Classes	PBP Type	Analytical Platform	References
1	Hydroxycinnamic acid glycosides, quercetin glycoside derivatives	Mountain papaya (*Vas concellea pubescens*)	LC-DAD-MS	[82]
2	Phenolics, myricetin hexoside, myricetin deoxyhexoside derivatives, quercetin hexoside, quercetin deoxyhexoside derivatives	Bayberries (*Myrica rubra Sieb. et Zucc*)	HPLC-DAD-ESI-MS	[83]
3	Simple phenolic and hydroxycinnamoylquinic acids and flavons, flavonols, flavanone and dihydrochalcone derivatives	Tomato (*Solanum lycopersicum*)	HPLC–ESI-QTOF	[84]
4	Anthocyanidins, aliphatic or aromatic acylated groups, sugar moieties	Eggplant (*Solanum melongena*); red leaf lettuce (*Lactuca sativa*); Pistachio (*Pistacia vera*) and others	HPLC-DAD-ESI-MS-MS	[85]
5	Proanthocyanidins, phenolic acids	Barley (*Hordeum vulgare*)	HPLC-DAD-MS	[86]

**Table 3:** Representative summary of recent studies reporting application of e-nose with or without other analytical platforms to evaluate flavor-associated metabolites in fresh PBPs.

Metabolites Class	PBP Used	Analytical Platform	Reference
Aldehydes, Alcohols and ketones	Apricots (*Prunus armeniaca)*	GC; e-nose; sensory analysis	[104]
Alcohols, terpene, aromatic hydrocarbons, aliphatic hydrocarbons	Mango (*Mangifera indica)*	GC; e-nose	[105]
Aromatic and aliphatic hydrocarbons	Blueberry (*Vaccinium corymbosum)*	e-nose	[106]
Alcohol, ester, aldehyde, terpenes	Grapes (*Vitis vinifera)*	GC; e-nose	[107]
Aldehydes, Alcohol, ketones	Tomato (*Lycopersicon esculentum)*	e-nose	[108]
Aldehydes, ketones, sulphur compounds, alkanes, terpenes, alcohols	Pineapple (*Ananus Comosus*)	e-nose	[109]
Acids, esters, Aldehydes, ketones, aliphatic and aromatic hydrocarbons	Citrus	GC-MS; e-nose	[110]
Ester, carboxylic acids, alcohols, Aldehydes, monterpenes	White and red fleshed peach (*Prunus persica*)	GC-MS; e-nose	[111]
Carboxylic acid, ester, alcohol,	Snake fruit (*Salacca zalacca*)	GC-MS; e-nose	[112]
Pyruvic acid	Onion (*Allium cepa*)	HPLC; e-nose	[113]
